# Why Radiomics Rarely Reaches the Clinic: Reproducibility, Validation, and Evidence Gap—A Critical Narrative Review

**DOI:** 10.3390/diagnostics16142266

**Published:** 2026-07-20

**Authors:** Jacopo Pozzi, Jacopo D’Argenzio, Serena Carriero, Maurizio Cè, Dario D’Arrigo, Pierpaolo Biondetti, Carolina Lanza, Salvatore Alessio Angileri, Matilde Pavan, Rossella Catona, Gianpaolo Carrafiello

**Affiliations:** 1 Postgraduate School in Radiodiagnostics, Università degli Studi di Milano, 20122 Milan, Italy; jacopo.dargenzio@unimi.it (J.D.); dario.darrigo@unimi.it (D.D.); matilde.pavan@unimi.it (M.P.); rossella.catona@unimi.it (R.C.); 2 Department of Diagnostic and Interventional Radiology, Fondazione IRCCS Ca’ Granda—Ospedale Maggiore Policlinico, Via Francesco Sforza 35, 20122 Milan, Italy; serena.carriero@gmail.com (S.C.); maurizioce.md1@gmail.com (M.C.); pierpaolo.biondetti@policlinico.mi.it (P.B.); carolina.lanza@unimi.it (C.L.); alessioangileri@gmail.com (S.A.A.); gianpaolo.carrafiello@unimi.it (G.C.); 3 Department of Oncology and Hemato-Oncology, Università degli Studi di Milano, 20122 Milan, Italy

**Keywords:** radiomics, reproducibility, clinical translation, external validation, methodological quality, meta-research, data leakage, reporting guidelines, clinical prediction models, evidence-based radiology

## Abstract

Radiomics has produced tens of thousands of publications yet almost no handcrafted radiomic signatures in routine clinical use, and the reasons are increasingly understood to be problems of reproducibility and clinical translation rather than of algorithms. This critical narrative review argues that the field systematically generates *paper-grade evidence*—findings sufficient to publish—far faster than *decision-grade evidence*—findings sufficient to change clinical practice. Drawing on meta-scientific research, we describe seven fragility mechanisms (publication bias, analytical flexibility, underpowering, HARKing [hypothesizing after the results are known], citation distortion, cognitive bias, and misaligned incentives) and show why radiomics is structurally exposed to all of them simultaneously: high-dimensional feature spaces, acquisition-dependent measurement instability, segmentation variability, retrospective single-centre data, small samples, and leakage-prone validation. We then summarise empirical evidence on the radiomics literature, which remains pervaded by suboptimal methodological quality, near-absent negative results, limited external validation, sparse calibration and clinical-utility assessment, low data and code sharing, and a measurable retraction signal. We interpret these patterns as the output of a self-reinforcing system rather than isolated errors, and argue that better algorithms alone cannot resolve them. Finally, we argue that closing this gap requires not better models but evidentiary discipline: the consistent, enforceable application of standards the field already has, and the calibration of published claims to the strength of the underlying evidence.

## 1. Introduction

Radiomics arose in the early 2010s from quantitative imaging and texture analysis research and was formalised in foundational accounts that reframed medical images as mineable, high-dimensional data [1,2,3]. A PubMed query for “radiomics” [All Fields] (PubMed/MEDLINE, searched May 2026) returned approximately 19,000 indexed records, of which roughly two-thirds were published in 2023 or later [4]. The premise is compelling: extracting hundreds to thousands of quantitative features from routine medical images to capture tumour biology non-invasively [1,2,5]. Each such feature or signature is, in effect, a candidate imaging biomarker, and its route to clinical use runs through the same qualification requirements that govern imaging biomarkers [6].However, this literature has produced virtually no tools in routine clinical use. Deep-learning detection, triage, and quantification systems have entered routine practice, with FDA authorisations of AI-enabled radiological devices exceeding 1000 by the end of 2025 [7]. However, no handcrafted radiomic signature has matched this deployment [8,9,10]. These deployments differ in kind: cleared deep-learning devices cluster in detection, triage, and quantification tasks, whereas handcrafted radiomic signatures are typically prognostic or predictive biomarker models, whose regulatory and deployment pathways diverge accordingly. The question is not whether radiomics works in principle but why a field generating thousands of papers per year has failed to produce decision-grade evidence—predictions robust enough to change clinical behaviour. We argue that this failure is not primarily algorithmic but evidentiary. We trace it to seven interacting fragility mechanisms (Section 3), show why radiomics is structurally exposed to them (Section 4), summarise their empirical signatures (Section 5), and set out the standards required to close the gap (Section 7 and Section 8).

This disconnect is not unique to radiomics; similar patterns appear across high-dimensional biomedical disciplines [11,12,13,14,15]. Meta-scientific research has identified structural mechanisms (publication bias, analytical flexibility, underpowered designs, and weak validation norms) that cause the literature to become populated with individually plausible but collectively unreliable findings [16,17,18,19]. Chalmers and Glasziou [20] famously estimated that a large share—on the order of 85%—of biomedical research investment may be avoidable waste; although the precise figure is debated, it conveys the scale of the concern. Smaldino and McElreath [21] formalised the key dynamic: when career success depends on publication productivity and publication depends on significant results, methods maximising publishability spread through the scientific population even if they degrade reliability. This could lead to literature in which *paper-grade evidence* can accumulate much faster than *decision-grade evidence*. We use *paper-grade evidence* to denote findings sufficient to support the publication of a radiomic association or model but insufficient to support clinical decision-making. Such evidence is typically retrospective, internally evaluated, discrimination-centred, and vulnerable to analytical flexibility. In contrast, *decision-grade evidence* denotes evidence sufficient to justify a change in clinical behaviour. Operationally, the model or signature must satisfy, at minimum: (i) independent external validation on data not used in development; (ii) explicit assessment and reporting of calibration; (iii) a pre-specified, analytically locked pipeline; (iv) benchmarking against clinically available or standard-of-care predictors; (v) demonstration of incremental value through decision-analytic methods such as net-benefit or decision-curve analysis; and, at implementation, (vi) prospective clinical evaluation [9,22,23,24]. This is a different axis from certainty grading. Schemas such as GRADE [25] assess how certain a body of evidence is about a defined clinical question; the paper-grade/decision-grade distinction asks whether a given model has been subjected to the tests above. The two can diverge: an association may rest on high-certainty evidence and still be paper-grade if its calibration, incremental value, and clinical utility have never been examined. This is not, however, a uniform characterisation. Programmes with standardised multicentric protocols, IBSI-compliant extraction, and independent external validation produce evidence of a substantially higher order than the typical retrospective single-centre study. The mechanisms we describe do not implicate individual researchers: they are properties of the research ecosystem, and they can produce fragile evidence even in the absence of misconduct [21,26].

## 2. Materials and Methods: Scope, Evidence Selection, and Narrative Synthesis

This review synthesises meta-scientific evidence on the conditions under which radiomics research is generated, validated, and translated into clinical practice. It is a critical narrative synthesis, reported in line with SANRA [27]; it interprets evidentiary mechanisms and their empirical signatures rather than pooling a pre-specified question, and its source selection is purposive rather than exhaustive.

Sources were identified through structured searches of PubMed/MEDLINE and Google Scholar, run through May 2026, combining the core term with methodological and translational qualifiers, and extended by backward and forward citation tracing of key methodological papers, meta-research studies, consensus documents, and reporting guidelines; the full search strategy and representative search strings are given in Supplementary Materials S1. We prioritised five categories of evidence: (i) radiomics-specific meta-research studies; (ii) systematic reviews and methodological audits assessing study quality, validation, reporting, reproducibility, or clinical translation; (iii) empirical studies quantifying specific failure modes such as leakage, acquisition dependence, feature instability, and insufficient sample size; (iv) consensus statements and reporting or appraisal frameworks relevant to radiomics, imaging AI, and clinical prediction modelling; and (v) foundational meta-scientific literature on publication bias, analytical flexibility, underpowered research, citation distortion, cognitive bias, and scientific incentives. Sources were included when they contributed directly to one of three analytic functions: defining a mechanism of evidentiary fragility, documenting an empirical signature of that mechanism in radiomics or imaging AI, or supporting a proposed governance response. Sources representing stronger or more successful translation—standardised multicentric programmes, IBSI-compliant extraction, externally validated signatures, and biomarker-qualification efforts—were deliberately sought and included alongside the critical literature.

## 3. Meta-Scientific Mechanisms of Evidentiary Fragility

Several mechanisms known from meta-scientific research can make biomedical literatures fragile, describing conditions under which plausible, statistically significant, or technically sophisticated findings may accumulate faster than robust, transportable, clinically useful evidence. They include publication bias [28,29,30,31], analytical flexibility and the garden of forking paths [32,33], underpowering and the winner’s curse [34,35,36], HARKing [37,38,39], citation distortion [40], and cognitive biases including motivated reasoning and confirmation bias [41,42].

**Figure 1:**
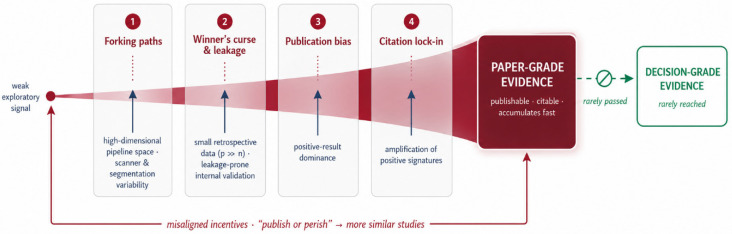
The self-reinforcing engine of fragile evidence. A weak exploratory signal (left) is progressively amplified as it passes through four interacting mechanisms, each shown with the general meta-scientific mechanism (upper label) and its radiomics-specific manifestation (lower label): analytical flexibility and the garden of forking paths, operating on a high-dimensional pipeline space with scanner- and segmentation-dependent measurement; the winner’s curse and data leakage, operating on small retrospective samples (*p* ≫ *n*) under leakage-prone internal validation; publication bias, which selects positive results; and citation lock-in, which amplifies positive signatures. The output is paper-grade evidence, which only rarely passes the gate to decision-grade evidence. Misaligned incentives close the loop (bottom), returning the system to further exploratory studies rather than to confirmatory ones. Notation: *p* ≫ *n*, indicating that the number of candidate predictors far exceeds the sample size.

These mechanisms are interrelated and can act as a self-reinforcing system (Figure 1). Publication bias rewards positive findings [28,31], which increases the payoff from analytical flexibility [32,33]. High-dimensional pipelines may amplify this: the larger the space of defensible analytic choices, the more paths can lead to a publishable result. Underpowering ensures that any significant finding overestimates the true effect [34,35]. Cognitive mechanisms—motivated reasoning [41], confirmation bias [42], and, in AI-assisted settings, automation bias [43]—guide researchers through the garden of forking paths toward configurations that yield significance, without deliberate deception. The resulting paper reports inflated performance. Citation distortion [40] can propagate these inflated claims; high-performing models are cited without reference to validation status, progressively hardening provisional findings into accepted facts. Finally, incentive structures determine which research behaviours are rewarded. When career advancement, publication success, and institutional prestige depend more on producing positive and novel outputs than on generating negative results, independent replications, or externally validated tools, methods that maximise publishability can spread even if they do not maximise reliability [21,26].

## 4. Why Radiomics Is Structurally Exposed

The general mechanisms described above find an extreme case in radiomics, a field in which every structural vulnerability is simultaneously present. A high-dimensional feature space, physically unstable measurements, predominantly small retrospective monocentric samples, and leakage-prone validation converge to maximise analytical flexibility and structurally weaken evidentiary discipline.

### 4.1. Combinatorial Analytical Flexibility

A standard radiomic pipeline extracts hundreds to thousands of features from segmented image regions [44,45,46]. Each subsequent step—feature selection, normalisation, classifier choice, and hyperparameter tuning—is individually defensible; in combination, however, these choices generate a vast combinatorial space of plausible workflows. In radiomics, this multiplicity can produce a “vibration of effects,” whereby materially different results emerge from the same data depending on how the analysis is specified. Such flexibility makes unstable or spurious associations increasingly easy to obtain unless the pipeline is rigorously constrained and analytical multiplicity is explicitly controlled [47]. Methodological variants further expand this space: delta-radiomics, for example, multiplies the configuration count by the number of time-points and delta-metric choices [48].

### 4.2. Acquisition-Dependent Measurement Instability

Radiomic features are computational derivatives of images that are themselves products of complex acquisition and reconstruction chains, and their values inherently depend on the technical conditions under which images are generated, including scanner platform, acquisition protocol, and voxel geometry [49,50,51,52]. Controlled CT experiments have shown that tube current, noise index, and iterative reconstruction levels substantially alter feature reproducibility, especially for texture features that depend on spatial intensity distributions [53]. This technical dependence persists in patient-level data: in a same-patient study of liver metastases reconstructed across multiple dose levels, section thicknesses, kernels, and reconstruction settings, only 11% of tested radiomic features remained reproducible across technical variations, with reconstructed section thickness producing the largest single-parameter effect [54]. Zhu et al. [55] tested 93 features across five CT systems: scan–rescan (test–retest) repeatability was excellent (97.1% repeatable), and intra-system reproducibility across dose levels was high (mean ICC = 0.945), but inter-system reproducibility was near zero (mean ICC = 0.157; 0% of features with ICC > 0.90). Zhang et al. [56] showed that on photon-counting detector CT, 0% of features were robust to slice-thickness changes. A systematic review of 481 studies confirmed that acquisition introduces more feature variability than segmentation, particularly for MRI [57]. Repeatability concerns extend beyond CT and MRI: a test–retest analysis of deep-learning-based PSMA-PET segmentation—cited here as a comparator—documented non-trivial inter-scan variability even when the segmentation algorithm was deterministic [58]. The Image Biomarker Standardisation Initiative (IBSI) Phase 1 [46] and Phase 2 [59] have standardised computational definitions but cannot eliminate the physical dependence on acquisition conditions. Statistical harmonisation methods such as ComBat can reduce inter-site variability [60,61], but their corrective capacity remains incomplete and context-dependent [61,62].

### 4.3. Segmentation Variability

Radiomic feature extraction often relies on manually delineated regions of interest; inter-reader variability can propagate directly into feature values and their stability, although standardisation recommendations now exist [63,64,65]. A meta-analysis of CT-based machine-learning studies in renal tumours further found that pooled diagnostic performance differed according to phase-selection and manual segmentation strategy, with contour-focused single-phase approaches showing the highest pooled AUC [66]. Automated and semi-automated segmentation, including deep-learning methods, can reduce inter-reader variability but introduces its own reproducibility considerations; even deterministic algorithms can show non-trivial inter-scan variability [58], and feature stability varies across segmentation methods [67].

### 4.4. Retrospective, Monocentric Data Dominance

In the 2023 NEVER study [68], 95% of 149 sampled publications were retrospective, 75% were single-centre, and 91% used private data; in the 2024 self-reporting meta-research [69], 94% were retrospective and 68% single-centre. Such designs are especially exposed to all preceding vulnerabilities: when development and evaluation data originate from the same institutional environment, they often share scanners, protocols, and local clinical workflows, creating a weak test of generalisability and increasing the risk that models capture centre-dependent regularities rather than transportable biological signal [70,71]. Overlapping or reused patient cohorts have also been documented across radiomics publications, further complicating the independence and cumulative interpretation of the evidence base [72,73].

### 4.5. Small Samples Relative to Analytic Complexity

Zhong et al. [74] examined 116 radiomics studies from seven leading journals published in 2023: only 9.5% justified their sample size, the median events per predictor parameter (EPP) was 7.5, and, under the Riley et al. criteria [36], only 10.3% had a sufficient training sample size, with a median deficit of 268 patients. Horvat, Papanikolaou, and Koh [75] further noted that fewer than 20 published radiomics studies had incorporated clinical trial data, and that none had prospectively implemented radiomics as a clinical decision-support tool.

### 4.6. Leakage-Prone Validation

Leakage is not a single error but a family of train–test contamination mechanisms. In radiomics, recurrent forms include: (1) feature selection performed on the full dataset before cross-validation; (2) oversampling procedures such as SMOTE applied before data splitting; (3) harmonisation fitted on the entire dataset rather than within the training loop; (4) preprocessing statistics, including normalisation parameters, estimated using all available observations; and (5) hyperparameter optimisation evaluated through non-nested cross-validation [76,77,78,79,80]. Kapoor and Narayanan [77] documented leakage in at least 294 papers across 17 scientific fields, showing how easily it can generate overoptimistic claims. Within imaging pipelines, Marzi et al. [79] demonstrated that harmonisation before data splitting itself creates leakage and inflates apparent performance, while Gidwani et al. [80] showed that inconsistent partitioning across normalisation, feature selection, hyperparameter selection, and model assessment can markedly idealise radiomic models. The magnitude of the bias is substantial: oversampling leakage inflated AUC by up to +0.343 and produced AUCs as high as 0.90 on random data [78], whereas feature-selection leakage increased AUC-ROC by up to +0.15 across ten public radiomics datasets [76]. Even transparent external validation can expose the fragility of internally optimised signatures: a PET-radiomics model for recurrence-site prediction after head-and-neck re-irradiation declined from a reported balanced accuracy of 84.5% to 70% in an independent cohort, recovering only partially to 78% after cut-off recalibration [81].

### 4.7. Weak Linkage to Clinical Decision-Making

Calibration assessment and clinical utility evaluation remain consistently underreported in RQS-based appraisals [82,83]. In HPV-status prediction for oropharyngeal cancer, only 5% of studies reported calibration statistics [84]; in MRI-radiomics studies for MGMT promoter methylation prediction in glioma, only 8% did so [72]. In contrast, discrimination-focused reporting was far more common: 89% of HPV studies reported discrimination statistics [84], and all MGMT studies reported AUC or accuracy [72]. However, even a model achieving a high internal AUC offers limited clinically actionable information if its calibration is unreported, its net benefit has not been assessed [85,86], and its performance has not been benchmarked against simpler or clinically available predictors [44].

## 5. Empirical Signatures of Evidentiary Fragility

**Figure 2:**
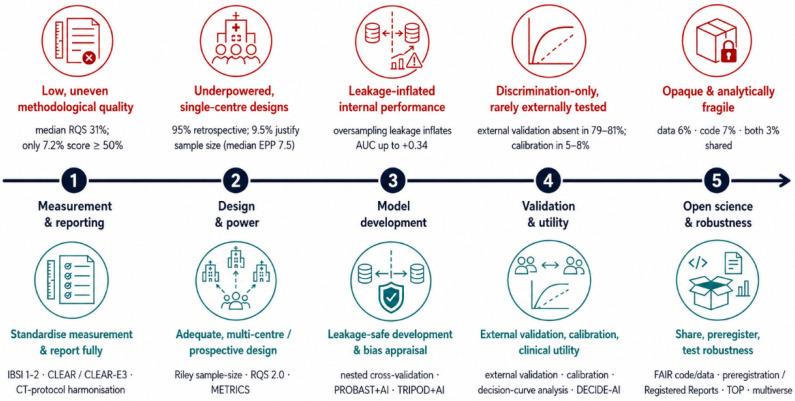
Fragility signatures, safeguards, and standards across the radiomics pipeline. Each of five sequential pipeline stages (centre) is paired with the empirical signature of paper-grade practice observed in the literature (above) and with the safeguards and standards that would address it (below): measurement and reporting—low, uneven methodological quality against IBSI Phases 1–2, CLEAR/CLEAR-E3, and CT-protocol harmonisation; design and power—underpowered, retrospective, single-centre designs against formal sample size calculation for prediction models, with study-level appraisal by RQS 2.0 and METRICS; model development—leakage-inflated internal performance against nested cross-validation, PROBAST + AI, and TRIPOD + AI; validation and utility—discrimination-centred evaluation that is rarely externally tested against independent external validation, calibration, decision-curve analysis, and DECIDE-AI; and open science and robustness—opaque and analytically fragile practice against FAIR code and data, preregistration and Registered Reports, TOP, and multiverse analysis. Values are indicative of the signatures reported in Table 1; the corresponding standards are set out in full in Table 2.

Across the literature, each fragility mechanism described above leaves a quantifiable trace. Table 1 collects these signatures alongside the mechanism each one reflects, and Figure 2 pairs them with the pipeline stage at which they arise and the safeguards that address them.

**Table 1:** Fragility mechanisms and their empirical signatures in radiomics. Rows are organised by fragility mechanism.

Mechanism of Evidentiary Fragility	Expected Ecosystem-Level Signature	Representative Measured Evidence in Radiomics
**1. Persistently low and uneven methodological maturity**	Formal standards, reporting frameworks, and quality tools expand, yet the average methodological quality of the published literature remains low and varies across radiomics subdomains.	Large-scale RQS evidence remains consistently poor: mean RQS 26.1% across 3258 assessments, with only 7.2% reaching ≥ 50% of the maximum score [83]; median RQS 31% across 1574 publications [82]; delta-radiomics median RQS 25%, with 51.2% of studies scoring < 25% [48]; endometrial MRI radiomics mean RQS 13.77 [87]
**2. Positive result selection and publication bias**	Null, negative, and non-superior findings are selectively underrepresented, while positive claims dominate the published record and are often insufficiently benchmarked against simpler alternatives.	Positive findings dominate the literature: NEVER found only 1/149 negative studies (0.7%) and no non-radiomic comparator in 44% [68]; glioma radiomics reported positive findings in 26/27 studies (96%) [88]; cardiovascular radiomics showed funnel-plot asymmetry by Egger’s test (z = −2.39, *p* = 0.017) [89].
**3. Leakage-prone analytical flexibility**	Researcher degrees of freedom and workflow errors that compromise separation between model development and evaluation inflate apparent performance.	Empirical leakage studies show substantial optimism: feature selection outside cross-validation inflated AUC by up to 0.15 [76]; oversampling before cross-validation biased AUC by up to 0.34, produced AUCs up to 0.90 with random outcomes, and was likely present in 5/34 radiomics papers from 2023 [78]
**4. Underpowered model development and winner’s curse**	Sample sizes remain too small relative to model complexity, favouring unstable estimates, optimistic effect sizes, and poor transportability.	In 116 binary-outcome radiomics prediction studies, only 11/116 (9.5%) justified sample size, and only 6 included an a priori calculation; median training size was 150, median EPP 7.5, median Riley-based shortfall 268 patients, and only 12/116 (10.3%) met all adequacy criteria [74].
**5. External-validation deficit and fragile generalisability**	Models are rarely tested on genuinely independent populations; when transported beyond development data, apparent performance commonly attenuates.	External validation was absent in 121/149 NEVER studies (81%) [68]; across 1574 radiomics publications, only 14% reported independent external validation, and 32% lacked any separate validation set [82]. In radiologic deep learning, external validation reduced median AUC by −0.046, with decline in 70/86 algorithms (81%) [90].
**6. Acquisition-driven measurement instability**	Apparent radiomic signal fails to transport across scanners, platforms, or acquisition settings, indicating vulnerability to non-biological measurement variation.	Acquisition effects markedly impair reproducibility: across five CT systems, 97.1% of features were repeatable on test–retest (scan–rescan), but inter-system reproducibility was poor (mean ICC/CCC 0.157 ± 0.174), with no feature reaching ICC/CCC > 0.90 across systems [55]; on photon-counting detector CT, no features were robust to high-pitch acquisition or slice-thickness changes [56]; and acquisition effects exceeded segmentation effects in a 481-study reliability review [57].
**7. Reporting opacity, self-assessment inflation, and weak open-science practice**	Methodological errors remain difficult to detect, formal self-audit is rare, and independent verification is constrained by incomplete reporting and limited sharing of code and data.	Transparency remains limited: only 7/117 studies (6%) included a self-reported checklist/quality score [69]; CLEAR adoption was 2%, with self-reported adherence exceeding expert-confirmed adherence by 21 percentage points [91]; among 257 studies, 6% shared data/open datasets, 7% shared code, and 3% shared both [92]; 0/195 empirical radiology articles shared analysis scripts [93].
**8. Quality-sensitive heterogeneity in evidence synthesis**	Methodological quality is not merely a descriptive deficit: it becomes a measurable source of variation in pooled performance estimates.	Study quality measurably affects pooled evidence: in endometrial MRI radiomics, higher RQS was associated with lower QUADAS-2 risk, more recent publication year, and higher reported performance [87]; and in CT hematoma-expansion deep learning, subgroup analyses showed significant performance differences by segmentation technique and study quality [94].
**9. Weak cumulative evidence and stalled clinical translation**	Local methodological fragilities accumulate into low certainty evidence at synthesis level and limited progression toward clinically embedded use.	Meta-evidence remains low-certainty: among 53 re-performed radiomics meta-analyses, only 3/53 associations (5.7%) were convincing, and 43/53 (81%) were weak [95]. Fewer than 20 oncologic radiomics studies used clinical-trial data, and no published model had been prospectively implemented as routine clinical decision support [75]; this is consistent with recent analyses describing a widening publication–translation gap [8].

Abbreviations: AUC, area under the receiver operating characteristic curve; CCC, concordance correlation coefficient; EPP, events per predictor parameter; ICC, intraclass correlation coefficient; RQS, Radiomics Quality Score (further abbreviations are defined in the Abbreviations list).

**Table 2:** Translational pipeline stages: vulnerabilities, minimum requirements, and standards. Rows follow the translational pipeline sequence.

Translational Stage	Principal Vulnerability/Failure Mode	Minimum Operational Requirement for a Non-Exploratory Clinical Claim	Primary Standard(s), Guideline(s), or Framework(s)	Empirical Evidence that the Requirement Remains Insufficiently Met
**1. Study conception**	Analytical flexibility, post hoc hypothesis shaping, selective reporting	Pre-specify the clinical question, population, endpoint, candidate predictors, analysis plan, validation strategy, and primary performance metrics	Preregistration [96]; Registered Reports [97]; TOP guidelines [98]	In broader meta-research (predominantly psychology), positive findings were reported in 44% of Registered Reports versus 96% of standard publication models [97,99]
**2. Imaging measurement, feature extraction, and software traceability**	Acquisition-, preprocessing-, filter-, and software-dependent feature instability	Use IBSI-compliant definitions; report acquisition, reconstruction, preprocessing, interpolation, discretisation, filters, extraction software, and software version	IBSI Phase 1, 2 [46,59]; documented open implementation: PyRadiomics [100]	Across photon-counting and dual-energy CT systems, mean inter-system ICC was 0.157, and no feature reached ICC > 0.90 at matched dose [55]. Feature values differed across radiomics software implementations [101]. Vendor-dependent quantitative CT differences were also reported outside radiomics [102].
**3. Sample size adequacy and study positioning**	Underpowered model development; unstable estimates; inflated apparent performance	Provide a formal sample size justification using prediction-model criteria	Riley et al. [36]	Sample size justification was absent in 90.5% of studies; only 10.3% met strict Riley-based criteria; median shortfall: 268 patients [74].
**4. Model development and leakage-safe internal validation**	Data leakage; optimistic bias; non-nested feature selection; misuse of resampling	Nest preprocessing, feature selection, resampling, hyperparameter tuning, and model selection within training folds; use leakage-safe internal validation.	Radiomic-signature safeguards [103]; nested cross-validation principles [104]; PROBAST + AI [105].	AUC inflation reached +0.34 when oversampling preceded cross-validation [78] and +0.15 when feature selection was applied before cross-validation [76].
**5. Reporting transparency of radiomics and AI methods**	Incomplete or non-verifiable pipeline reporting	Report item-by-item against the framework appropriate to the study scope: CLEAR, CLEAR-E3, TRIPOD + AI, and CLAIM where applicable	CLEAR [44]; CLEAR-E3 [106]; TRIPOD + AI [23]; CLAIM 2024 [107]	Only 7/117 radiomics papers (6%) included a self-reported reporting checklist or quality-scoring document [69]. CLEAR adoption reached 2%; self-reported versus expert-confirmed adherence was 91% vs. 66% (mean gap, 21 percentage points) [91].
**6. Methodological appraisal**	Conflation of reporting quality, methodological quality, and translational maturity	Use structured tools for methodological appraisal and translational readiness, rather than relying on discrimination metrics or narrative claims alone	METRICS [45]; RQS 2.0 and Radiomics Readiness Levels [22]	Median RQS was 31% of the maximum across 1574 publications [82]. In a 2025 diagnostic-accuracy synthesis, study quality assessed with METRICS emerged as a significant source of between-study differences in subgroup analyses [94].
**7. Open science and computational reproducibility**	Unavailable code; inaccessible datasets; non-reproducible computational workflows	Share code and data where feasible; otherwise, state access restrictions and provide sufficient computational detail for independent re-analysis	FAIR principles [108]; TOP guidelines [98]	In 257 radiomics papers published in leading journals, only 6% shared data, and 7% shared code [92]. Private data were used in 91% of papers in the NEVER study [68] and 89% in a separate meta-research sample [69]. In broader radiology and nuclear medicine AI, only 1/161 private-data studies shared the dataset [109].
**8. External validation and transportability**	Internal-only evidence; poor transportability across institutions, scanners, and protocols	Perform external validation on independent data; distinguish internal, internal–external, and external validation	Validation hierarchy and clinical prediction-model guidance [110,111]	External validation was absent in 81% [68] and 79% [69] of radiomic studies; only 14% of 1574 publications included external validation [82].
**9. Calibration, clinical benchmarking, and incremental value**	Discrimination-only evaluation; absent calibration; untested incremental clinical value	Report calibration alongside discrimination; compare against clinical, non-radiomic, or standard-of-care baselines; quantify added value	Calibration principles [85,112]; CLEAR comparison requirements [44]	Calibration was reported in 1/19 HPV-prediction studies [84] and 2/26 MGMT-prediction studies [72]. 44% of radiomics studies included no non-radiomic comparator [68]. In prostate radiotherapy, adding MRI radiomics increased the C-index from 0.69 to 0.70 over a clinical model [113].
**10. Early clinical evaluation and trial-level evidence**	Retrospective performance claims substituted for early clinical evaluation; incomplete protocol and trial reporting	Evaluate live clinical performance, safety, workflow effects, and human-factor consequences before trial-level claims; use AI-specific reporting extensions for interventional protocols and trial reports	DECIDE-AI [24]; SPIRIT-AI [114]; CONSORT-AI [115]	In oncology AI, median SPIRIT-AI concordance was 78.2% across 12 RCT protocols [116]; median combined CONSORT 2010/CONSORT-AI concordance was 82% across 57 RCT reports [117]
**11. Deployment governance, regulatory readiness, and real-world translation**	Clinical-readiness claims without deployability, lifecycle governance, or regulatory fitness	Address trustworthiness, robustness, fairness, explainability, traceability, post-deployment monitoring, and applicable regulatory requirements before claiming clinical readiness	FUTURE-AI [118]; GMLP/IMDRF [119]; applicable medical-device regulation, including EU MDR where relevant [120]	The research–clinical translation gap has been described as widening [8]. Routine oncologic implementation remains limited [9,73]. Progress in cost-effectiveness analysis is minimal or insignificant across radiomics studies [83].

Abbreviations: AUC, area under the receiver operating characteristic curve; CCC, concordance correlation coefficient; EPP, events per predictor parameter; ICC, intraclass correlation coefficient; RQS, Radiomics Quality Score (further abbreviations are defined in the Abbreviations list).

### 5.1. Quality, Publication Bias, and Validation

Two large 2024–2025 evidence syntheses converge on the finding that radiomics study quality has improved over time but remains persistently low overall [82,83]. These concerns had already been documented by Park et al. in 2020 [121] and were subsequently reinforced by Spadarella et al. [122] in 2023. Using the Radiomics Quality Score (RQS), Kocak et al. [82] found a median RQS of 31% across 1574 unique publications, with a strong positive temporal trend (Kendall’s tau = 0.908, *p* < 0.001); and Barry et al. [83] reported a mean RQS of 26.1% ± 17.8% across 3258 RQS assessments (with only 7.2% reaching ≥ 50% of the maximum score). Complementary METRICS (METhodological RadiomICs Score [45], with an explanation-and-elaboration companion [123]) audits show that methodological quality remains, at best, moderate across several subfields: prostate MRI, 52% [124]; cardiac CT/MRI, 54.5% [89]; and glioma radiomics, 57% [88]. The cardiovascular METRICS audit also illustrates why reported performance should not be equated with evidentiary robustness. Despite a pooled AUC of 0.81, Cavallo et al. reported high heterogeneity, statistically significant funnel-plot asymmetry, and only moderate average methodological quality, with 9 papers eligible for the meta-analysis [89]. Direct evidence of positive-result bias comes from the NEVER meta-research study; only 1 of 149 radiomics articles published in Q1 clinical radiology journals reported negative results (0.7%; rounded to 1% by the authors) [68]. Consistently, a glioma radiomics synthesis found that 26 of 27 studies (96%) reported positive effects, which the authors interpreted as evidence of high non-statistical publication bias [88]. Across two independent radiomics meta-research samples, external validation was absent in 81% (121/149) [68] and 79% (93/117) [69] of studies. In the broader radiologic deep-learning literature (invoked here as a comparator to handcrafted radiomics), externally validated algorithms also frequently lose performance. Yu et al. [90] found at least some external performance decrease in 70 of 86 algorithms (81%). Zhong et al. [95] re-performed 53 meta-analyses; only 3 of 53 (5.7%) reached a convincing level of evidence, whereas 43 of 53 (81%) were rated as weak. A recent deep-learning diagnostic synthesis likewise paired QUADAS-2 with METRICS in its quality appraisal; subgroup analyses showed significant performance differences according to study quality, indicating that methodological rigor can materially influence pooled estimates [94].

### 5.2. Reporting Transparency

Transparency remains exceptional rather than routine in radiomics research. Among 117 radiomics papers, only 7 (6%) reported the use of any checklist or quality-scoring instrument [69], and adoption of the CLEAR (CheckList for EvaluAtion of Radiomics research) guideline across eligible literature has reached only 2% [91]. In 257 radiomics studies published in leading radiology journals, 16 (6%) shared data or used publicly available datasets, 20 (7%) shared code, and only 7 (3%) did both [92]; in the broader radiology and nuclear medicine AI literature, just 1 of 161 private datasets was made available [109]. Reproducibility is further constrained by software dependence; nominally equivalent radiomic features can differ materially across extraction packages because of discrepancies in preprocessing and implementation [101], a broader problem in quantitative imaging, as illustrated for instance by reported cross-platform differences in cardiac CT measurements [102]. The IBSI framework [46,59] provides the technical basis for comparability through consensus definitions, reference values, and benchmarking; however, reporting and transparency practices have not kept pace [91].

### 5.3. Benchmarks and Translation

The translational deficit is also reflected in how radiomics claims are benchmarked. In a meta-research analysis of radiomics studies published in leading radiology journals, 44% made no comparison with non-radiomic approaches [68]. In treatment-response radiomics for non-small-cell lung cancer, comparison with the current gold standard was absent in all but two studies [125]. Even when direct comparisons are performed, incremental value is often limited or absent: in prostate radiotherapy, adding MRI radiomics increased the C-index only from 0.69 to 0.70 relative to a clinical-only model [113]; in advanced melanoma, CT radiomics failed to improve on a simpler clinical model for predicting benefit from checkpoint inhibitors [126]; and in locally advanced rectal cancer, MRI-based radiomic models showed no definite added value over clinical models for predicting pathological complete response after neoadjuvant chemoradiotherapy [127]. The problem, therefore, is not simply that radiomics has been slow to reach clinical practice, but that much of the literature still stops short of establishing why practice should change. This is the translational gap described by Kocak, Pinto dos Santos, and Dietzel [8], and it persists despite measurable improvements in formal study quality: Barry et al. showed that the dimensions most consequential for clinical adoption remain among the least developed [83]. RQS 2.0 and its radiomics readiness levels were introduced precisely to make that gap visible, shifting appraisal from isolated methodological adequacy to staged translational maturity [22,128].

### 5.4. Retractions as an Ecosystem Signal

Retractions provide a complementary ecosystem-level indicator of evidentiary fragility in radiomics. Demircioğlu identified 93 retracted radiomics publications across six databases, corresponding to an estimated mean retraction rate of 6.7 per 10,000 publications—a rate that, in absolute terms, is comparable to or below several biomedical baselines and is therefore best read as a weak, indirect signal. Among the 20 cases examined in detail, 11 retraction notices (55%) did not clearly distinguish misconduct from error or assign responsibility, and no major radiological or oncological journal appeared to have retracted a radiomics publication [129]. Against a background of persistently low code- and data-sharing rates, which hinder independent scrutiny of published work [92], this pattern suggests that formal retractions may underestimate the broader burden of problematic radiomics studies [129].

## 6. From Recurrent Patterns to Systemic Interpretation

Section 3, Section 4 and Section 5 described fragility mechanisms, their structural amplification in radiomics, and their empirical signatures. We interpret these not as independent issues, but as the coupled components of a self-reinforcing system (Figure 1). Where Ioannidis [17] distinguishes useful from merely publishable research, Huang et al. [9] specify the criteria for clinical translation, and RQS 2.0 [22] grades study-level readiness. The paper-grade/decision-grade distinction locates the gap in the interaction between fragility mechanisms and the publication ecosystem rather than in any single study.

### Paper-Grade Evidence as the Product of a Self-Reinforcing System

Radiomics is structurally exposed because the vulnerabilities described above do not merely coexist; high dimensionality and analytical flexibility, acquisition- and segmentation-dependent measurement, retrospective single-centre designs, small samples, leakage-prone workflows, and limited sharing of code and data tend to co-occur and may jointly lower the threshold for exploratory findings to become publishable before they become clinically reliable [3,59,67,74,76,78,83,92,130,131,132,133,134,135].

Radiomic pipelines create many defensible analytical routes; preprocessing, discretisation, harmonisation, feature selection, classifier choice, hyperparameter tuning, and validation design can each be varied in ways that may appear individually reasonable. In such a setting, cognitive mechanisms such as motivated reasoning and confirmation bias may influence which routes are explored, retained, or interpreted as most compelling, without requiring deliberate misconduct [41,42]. When positive or apparently high-performing results are more likely to enter the visible literature, this flexibility can favour the accumulation of attractive findings over the accumulation of robust evidence, as illustrated in radiomics by the scarcity of negative studies [68].

Once published, these findings can acquire cumulative force, as provisional radiomic claims are transmitted through a literature already skewed toward positive findings rather than through a balanced evidentiary record. Broader meta-research shows that positive or statistically significant findings are preferentially cited, while citation networks can further transform qualified claims into apparent authority [40,136,137]. In the radiomics literature already enriched for positive results [68], this may create a secondary amplification layer; internally promising models can become part of the cumulative narrative before their external validity, calibration, and clinical utility have been adequately established. The same process is reinforced by institutional and publication incentives. Systems that reward novel, positive, technically sophisticated, and publishable outputs more strongly than correction, replication, external validation, or negative evidence can select for research behaviours that maximise publication success even when they do not maximise reliability [21,26].

The result is not literature of false claims, but literature in which paper-grade evidence can become visible, citable, and cumulatively influential before the harder tests required for decision-grade evidence have been satisfied [21,40,41,42,68]. Paper-grade evidence—typically retrospective, internally validated, and discrimination-centred—may justify publication but not clinical action. Decision-grade evidence requires independent external validation [9,75], adequate calibration [85], comparison with clinical alternatives, and incremental value demonstrated through decision-analytic evaluation [86,138]. Claim–evidence alignment should discipline the language of radiomics translation: exploratory studies should remain explicitly exploratory; internally validated models should be framed as candidates for external testing; generalisability should not be claimed without external validation; and clinical usefulness should not be claimed without calibration, benchmarking against clinically available alternatives, incremental value, and decision-analytic assessment of clinical utility—otherwise limited evidence is converted into overstatement, as described in the literature on biomedical spin, diagnostic test overinterpretation, and unsupported AI performance claims [139,140,141].

## 7. Standards and Safeguards Across the Radiomics Pipeline

Because the fragility described above is a property of the ecosystem rather than of any algorithm, the response is methodological discipline applied sequentially across the radiomics pipeline—from measurement and model development to validation and open science (Figure 2; Table 2).

### 7.1. Measurement, Reporting, and Statistical Planning

Stable measurement is the first requirement for credible radiomics. IBSI’s original feature-standardisation effort established consensus definitions and reference values for 169 radiomic features [46], while the subsequent filter-standardisation initiative provided reference implementations and verification resources for commonly used convolutional filters [59]. Measurement stability also depends on upstream image acquisition. Although not radiomics-specific, professional society efforts to harmonise CT protocols, such as the SIRM position papers [142,143], address an acquisition layer that is directly relevant to radiomic feature robustness, given the well-documented sensitivity of CT radiomics to scanner, reconstruction, slice-thickness, and discretisation choices [50,51,52]. Reporting standards are equally essential. The CLEAR guideline requires authors to report the software, version, feature definitions, segmentation and preprocessing details, and extraction parameters needed to assess reproducibility [44]; CLEAR-E3 provides worked explanations and examples for applying these requirements consistently [106]. Statistical planning must then match the effective complexity of the model development process. Riley et al. [36] provide practical methods for sample size calculation in prediction model development, and radiomics analyses should accordingly align candidate model complexity with the information content of the available data.

Radiomics ultimately produces candidate imaging biomarkers, whose clinical acceptance is judged within biomarker qualification frameworks. The FDA-NIH BEST resource defines biomarker categories and the goal of a qualified biomarker for a defined context of use [6], while the RSNA Quantitative Imaging Biomarkers Alliance (QIBA) issues Profiles that specify technical performance claims and conformance requirements for quantitative imaging biomarkers—for example, the DSC-MRI Profile for gliomas [144]. IBSI standardises upstream feature definitions [46,59], whereas these frameworks address the metrological and regulatory layer, which handcrafted radiomics has rarely engaged. Table 3 summarises the principal appraisal, reporting, and standardisation instruments and their differing scopes.

### 7.2. Leakage-Free Model Development

Data-dependent operations must be confined to the training process and re-estimated independently within each resampling fold or validation split. Radiomics-specific studies have quantified the inflation caused by feature selection leakage [76] and by oversampling before cross-validation [78], while broader machine-learning methodology has documented leakage as a pervasive source of irreproducible scientific claims [77]. Leakage can arise at multiple stages of the pipeline, including preprocessing, feature selection, resampling, harmonisation, and model or hyperparameter selection. The radiomics literature already provides direct empirical evidence for some of these mechanisms [76]; separate work in medical imaging has shown that harmonisation performed before data splitting can also induce leakage and inflate downstream performance estimates [79]. Nested cross-validation helps separate model tuning from model evaluation [104]. PROBAST + AI [105] and TRIPOD + AI [23] should then be used to assess risk of bias and to ensure complete reporting of the resulting prediction model study.

### 7.3. Validation Hierarchy, Calibration, and Clinical Utility

Independent external validation is the minimum requirement for claims of generalisability beyond the source data, and prospective clinical evaluation becomes increasingly important as tools approach implementation [110,111]. For systems entering early live clinical evaluation, DECIDE-AI provides stage-specific reporting guidance tailored to AI-based decision-support studies [24]. Discrimination alone is insufficient for clinical credibility. For studies making clinical applicability claims, calibration should be assessed and reported explicitly [85,112], and decision-analytic utility should be evaluated through net-benefit approaches such as decision-curve analysis [86,146]. These model-centred assessments should be complemented by clinically meaningful and patient-centred outcome measures, which remain less frequently foregrounded in the evaluation literature on AI in radiology [147].

### 7.4. Open Science and Preregistration

Reproducibility requires preservation and, where feasible, sharing of the analytic materials needed to interrogate a radiomics claim: code, extraction configurations, model specifications, and reusable data resources [92,98,108]. The current literature remains far from this standard. In two independent radiomics meta-research samples, 91% and 89% of studies relied on private data [68,69], while a dedicated audit of sharing practices identified limited availability of models, code, and datasets as a major obstacle to clinical translation [92]. Preregistration addresses a complementary vulnerability: the blurring of confirmatory and exploratory analysis. By fixing hypotheses and analysis plans before outcomes are inspected, it helps distinguish prediction from postdiction and limits the scope for selective analytical adaptation [96,148].

### 7.5. Testing Robustness to Analytic Flexibility

Reporting standards document the analytic pipeline that was chosen, but they cannot establish whether a result reflects a stable signal or only one path through the space of defensible choices. Multiverse and specification curve analyses make that dependence measurable; the model is re-estimated across the full set of reasonable specifications (discretisation, harmonisation, feature selection, and classifier), and the distribution of results is reported in place of a single pipeline [149], while the specification curve orders these fits to show how far the signature depends on any individual choice [150]. This converts the vibration of effects from an unmeasured threat into a reported quantity; a result stable across this space is credible, while one that emerges only under a narrow configuration might be an artefact of analytic flexibility.

### 7.6. The Shifting Technical Frontier: Foundation Models and Generative AI

Self-supervised imaging foundation models are the most credible technical answer to handcrafted radiomics’ small samples and weak transportability [151], yet they relocate rather than remove the problem, concentrating capability and raising validation and governance challenges that, by their developers’ own account, strain current medical AI evaluation [152]. Generative methods are similarly double-edged; synthetic-data augmentation and harmonisation can enlarge scarce datasets and attenuate scanner effects [153], but in controlled phantom data image-level generative harmonisation improved appearance while reducing radiomic feature stability [154], so such steps must be validated on the downstream radiomic endpoint, not assumed beneficial.

## 8. Implications for the Field: From Available Standards to Routine Adoption

Although a substantial methodological infrastructure now exists [155,156,157] and formal quality has improved, its uptake remains marginal (Section 5.2). This reflects not the absence of standards but an incentive asymmetry. Methodological safeguards impose immediate costs on individual investigators (time, expertise, larger samples, and reduced analytical flexibility), while their benefits accrue collectively, through more reliable cumulative knowledge. The result is a collective action problem, mapping onto the distinction between the rewards of getting work published and those of getting it right [158]; as long as publication and advancement track productivity and positive findings, the privately rational choice diverges from the collectively useful one. Compliance will not become routine until methodological rigour is made consequential at the point of publication and evaluation [96,158].

Building on this incentive logic [158], reform should follow four design principles: interventions should be structural rather than exhortatory, composable rather than monolithic, enforceable rather than voluntary, and incentive-compatible rather than virtue-demanding. These principles are proposals, not validated remedies. Their effect on radiomics translation, as distinct from reporting quality, has not been tested, and each can fail in predictable ways. Entry requirements may favour groups already equipped to meet them, and mandates may produce formal rather than substantive compliance—a pattern already suggested by the divergence between self-reported and expert-confirmed CLEAR adherence [91]. They are accordingly best assessed against the outcome indicators set out below.

The principles translate into distinct responsibilities. Authors should pre-specify analytic plans, report against CLEAR, share code and data where feasible, and calibrate conclusions to the evidentiary tier actually reached [96,98,106,108,148]. Reviewers should apply PROBAST + AI and METRICS and assess whether a study’s claims match that tier [45,105]. At the editorial level, screening submissions against minimum methodological requirements before peer review, and weighing whether a study’s claims are commensurate with the evidence it attains, would serve the same end [22]. Registered Reports are the most promising structural lever for confirmatory work. In psychology, the only field where the format has been evaluated at scale, they reduced positive first hypothesis results from 96% of standard reports to 44% [97,99]—an effect not yet tested in radiomics, but one that shows how decoupling publication from result direction reshapes the visible evidence base. Funders should require data-sharing and, where appropriate, preregistration as conditions of award [96,98,108], and institutions should reward rigour and transparency over venue prestige, in line with DORA [159]. Progress should be tracked against concrete indicators: a rising share of published negative results, higher median quality scores, routine external validation, and greater use of Registered Reports for confirmatory studies.

## 9. Limitations and Boundary Conditions

Several limitations bound the claims of this review. By design it is a critical narrative synthesis with purposive, question-driven source selection rather than a systematic, bias-controlled survey; it is therefore itself exposed to the selection effects it describes, and its conclusions are interpretive rather than quantitative. The supporting evidence is also of uneven provenance: some is direct, such as the controlled leakage-inflation experiments; some is indirect, such as the retraction signal; and some is imported by analogy from deep-learning and other high-dimensional fields. The evidence base is also concentrated, as much of the appraisal infrastructure underlying the quality critique is derived from a small number of investigators and groups; common provenance may therefore carry common assumptions, and the literature’s apparent convergence rests on a comparatively narrow foundation. In addition, most of the empirical signatures are snapshots of the 2020–2025 period, whereas most of the standards against which they are read (CLEAR, METRICS, TRIPOD + AI, RQS 2.0) postdate much of the literature they appraise. Part of the observed under-adoption is therefore temporal, and this review necessarily describes the field with a delay, understating improvements already under way.

Moreover, robust work at the frontier of the field is not in dispute. Where formal certainty grading has been applied, it finds real signal. An overview that re-performed 53 radiomics meta-analyses graded three associations as convincing and seven as highly suggestive [95], and an umbrella review of artificial intelligence in cancer imaging rated roughly one-third of pooled estimates as moderate certainty [160]. Methodological quality is also rising over time [82,83], and a recent field review reaches the same diagnosis as this one; reproducibility and external validation, not algorithmic novelty, are the rate-limiting steps for clinical adoption, though standardisation efforts are beginning to address them [161]. Decision-grade radiomics therefore exists—produced by standardised, externally validated programmes that sit well above the typical retrospective single-centre study.

Finally, the entire translation gap is not attributable to evidentiary fragility. Imaging biomarker translation is intrinsically slow and capital-intensive, and its regulatory frameworks are still being consolidated. The European Union Artificial Intelligence Act phases in obligations for high-risk medical AI through 2026–2027 [162], and the United States Food and Drug Administration finalised its predetermined change control guidance only in late 2024 [163]. Limited data-sharing is often driven by privacy and governance constraints rather than reluctance, and for some clinical endpoints, the incremental value of radiomics over established predictors is genuinely marginal, itself a substantive finding and not a failure of effort.

## 10. Conclusions

Radiomics does not suffer primarily from a shortage of algorithms or features; it suffers from an evidentiary architecture in which paper-grade findings accumulate faster than the decision-grade evidence required to change clinical practice. The mechanisms responsible—analytical flexibility, measurement instability, underpowered and single-centre designs, leakage-prone validation, selective publication, and incentive misalignment—are mutually reinforcing, which is why purely technical fixes have not closed the translational gap. The constructive implication is that the field already possesses most of the standards it needs; what is missing are the composable, enforceable, and incentive-compatible mechanisms that make their sequential application routine and consequential, together with the editorial norm that claims be matched to the evidentiary tier actually achieved. Realigning publication with reliability is the path from paper-grade to decision-grade radiomics.

## Figures and Tables

**Table 3:** Appraisal, reporting, and standardisation instruments relevant to radiomics.

Instrument	Function	Scope	Body	Ref.
**IBSI (Phase 1–2)**	Measurement standardisation	Radiomic features	IBSI	[46,59]
**CLEAR (+CLEAR-E3)**	Reporting checklist	Radiomics studies	ESR/EuSoMII	[44,106]
**METRICS (+METRICS-E3)**	Methodological-quality score	Radiomics studies	EuSoMII	[45,123]
**RQS 2.0 + RRL**	Quality + translational readiness	Radiomics studies	RQS group	[22]
**TRIPOD + AI**	Reporting guideline	Prediction models (regression/ML)	TRIPOD/EQUATOR	[23]
**PROBAST + AI**	Risk-of-bias & applicability	Prediction models (regression/ML)	PROBAST group	[105]
**CLAIM (2024)**	Reporting checklist	AI in medical imaging	Radiology: AI	[107]
**QIBA Profiles**	Metrological performance claims & conformance	Quantitative imaging biomarkers	RSNA QIBA	[145]
**BEST**	Biomarker taxonomy & qualification	Imaging/non-imaging biomarkers	FDA–NIH	[6]

## Data Availability

No new data were created or analysed in this study. Data sharing is not applicable to this article.

## Supplementary Materials

The following supporting information can be downloaded at: https://www.mdpi.com/article/10.3390/diagnostics16142266/s1, Supplementary S1: Search strategy.

## Abbreviations

AUC, area under the receiver operating characteristic curve; BEST, Biomarkers, EndpointS, and other Tools; CCC, concordance correlation coefficient; C-index, concordance index; CI, confidence interval; CLAIM, Checklist for Artificial Intelligence in Medical Imaging; CLEAR, CheckList for EvaluAtion of Radiomics research; CONSORT-AI, Consolidated Standards of Reporting Trials–AI extension; CT, computed tomography; DECIDE-AI, reporting guideline for early clinical evaluation of AI decision-support; DORA, Declaration on Research Assessment; DSC-MRI, dynamic susceptibility contrast MRI; EPP, events per predictor parameter; EU MDR, European Union Medical Device Regulation; FAIR, Findable, Accessible, Interoperable, and Reusable; FDA, U.S. Food and Drug Administration; GAN, generative adversarial network; GMLP, Good Machine Learning Practice; GRADE, Grading of Recommendations Assessment, Development and Evaluation; HARKing, hypothesising after the results are known; HPV, human papillomavirus; IBSI, Image Biomarker Standardisation Initiative; ICC, intraclass correlation coefficient; IMDRF, International Medical Device Regulators Forum; IQR, interquartile range; METRICS, METhodological RadiomICs Score; MGMT, O6-methylguanine-DNA methyltransferase; MRI, magnetic resonance imaging; NSCLC, non-small-cell lung cancer; PET, positron emission tomography; PROBAST + AI, Prediction model Risk Of Bias ASsessment Tool–AI extension; PSMA, prostate-specific membrane antigen; QIBA, Quantitative Imaging Biomarkers Alliance; QUADAS-2, Quality Assessment of Diagnostic Accuracy Studies 2; Q1/Q3, first/third quartile; rCBV, relative cerebral blood volume; ROC, receiver operating characteristic; RQS, Radiomics Quality Score; RRL, Radiomics Readiness Levels; SANRA, Scale for the Assessment of Narrative Review Articles; SMOTE, Synthetic Minority Oversampling Technique; SPIRIT-AI, Standard Protocol Items: Recommendations for Interventional Trials–AI extension; TOP, Transparency and Openness Promotion; TRIPOD + AI, Transparent Reporting of a multivariable prediction model for Individual Prognosis Or Diagnosis–AI extension.

## References

[1] Lambin P., Leijenaar R.T.H., Deist T.M., Peerlings J., de Jong E.E.C., van Timmeren J., Sanduleanu S., Larue R.T.H.M., Even A.J.G., Jochems A. (2017). Radiomics: The bridge between medical imaging and personalized medicine. Nat. Rev. Clin. Oncol..

[2] Gillies R.J., Kinahan P.E., Hricak H. (2016). Radiomics: Images Are More than Pictures, They Are Data. Radiology.

[3] Kumar V., Gu Y., Basu S., Berglund A., Eschrich S.A., Schabath M.B., Forster K., Aerts H.J.W.L., Dekker A., Fenstermacher D. (2012). Radiomics: The process and the challenges. Magn. Reson. Imaging.

[4] Kocak B., Baessler B., Cuocolo R., Mercaldo N., Pinto Dos Santos D. (2023). Trends and statistics of artificial intelligence and radiomics research in Radiology, Nuclear Medicine, and Medical Imaging: Bibliometric analysis. Eur. Radiol..

[5] Ferrari R., Trinci M., Casinelli A., Treballi F., Leone E., Caruso D., Polici M., Faggioni L., Neri E., Galluzzo M. (2024). Radiomics in radiology: What the radiologist needs to know about technical aspects and clinical impact. Radiol. Med..

[6] FDA-NIH Biomarker Working Group (2016). BEST (Biomarkers, EndpointS, and Other Tools) Resource.

[7] U.S. Food and Drug Administration, Center for Devices and Radiological Health (2026). Artificial Intelligence-Enabled Medical Devices [Internet].

[8] Kocak B., Pinto Dos Santos D., Dietzel M. (2025). The widening gap between radiomics research and clinical translation: Rethinking current practices and shared responsibilities. Eur. J. Radiol. Artif. Intell..

[9] Huang E.P., O’Connor J.P.B., McShane L.M., Giger M.L., Lambin P., Kinahan P.E., Siegel E.L., Shankar L.K. (2023). Criteria for the translation of radiomics into clinically useful tests. Nat. Rev. Clin. Oncol..

[10] Limkin E.J., Sun R., Dercle L., Zacharaki E.I., Robert C., Reuzé S., Schernberg A., Paragios N., Deutsch E., Ferté C. (2017). Promises and challenges for the implementation of computational medical imaging (radiomics) in oncology. Ann. Oncol..

[11] Ioannidis J.P.A., Ntzani E.E., Trikalinos T.A., Contopoulos-Ioannidis D.G. (2001). Replication validity of genetic association studies. Nat. Genet..

[12] Varoquaux G., Cheplygina V. (2022). Machine learning for medical imaging: Methodological failures and recommendations for the future. npj Digit Med..

[13] Errington T.M., Mathur M., Soderberg C.K., Denis A., Perfito N., Iorns E., Nosek B.A. (2021). Investigating the replicability of preclinical cancer biology. eLife.

[14] Roberts M., Driggs D., Thorpe M., Gilbey J., Yeung M., Ursprung S., Aviles-Rivero A.I., Etmann C., McCague C., Beer L. (2021). Common pitfalls and recommendations for using machine learning to detect and prognosticate for COVID-19 using chest radiographs and CT scans. Nat. Mach. Intell..

[15] Carp J. (2012). On the Plurality of (Methodological) Worlds: Estimating the Analytic Flexibility of fMRI Experiments. Front. Neurosci..

[16] Ioannidis J.P.A. (2005). Why Most Published Research Findings Are False. PLoS Med..

[17] Ioannidis J.P.A. (2016). Why Most Clinical Research Is Not Useful. PLoS Med..

[18] Munafò M.R., Nosek B.A., Bishop D.V.M., Button K.S., Chambers C.D., Percie Du Sert N., Simonsohn U., Wagenmakers E.J., Ware J.J., Ioannidis J.P.A. (2017). A manifesto for reproducible science. Nat. Hum. Behav..

[19] Van Calster B., Steyerberg E.W., Wynants L., Van Smeden M. (2023). There is no such thing as a validated prediction model. BMC Med..

[20] Chalmers I., Glasziou P. (2009). Avoidable waste in the production and reporting of research evidence. Lancet.

[21] Smaldino P.E., McElreath R. (2016). The natural selection of bad science. R Soc. Open Sci..

[22] Lambin P., Woodruff H.C., Mali S.A., Zhong X., Kuang S., Lavrova E., Khan H., Lekadir K., Zwanenburg A., Deasy J. (2025). Radiomics Quality Score 2.0: Towards radiomics readiness levels and clinical translation for personalized medicine. Nat. Rev. Clin. Oncol..

[23] Collins G.S., Moons K.G.M., Dhiman P., Riley R.D., Beam A.L., Van Calster B., Ghassemi M., Liu X., Reitsma J.B., van Smeden M. (2024). TRIPOD+AI statement: Updated guidance for reporting clinical prediction models that use regression or machine learning methods. BMJ.

[24] Vasey B., Nagendran M., Campbell B., Clifton D.A., Collins G.S., Denaxas S., Denniston A.K., Faes L., Geerts B., Ibrahim M. (2022). Reporting guideline for the early-stage clinical evaluation of decision support systems driven by artificial intelligence: DECIDE-AI. Nat. Med..

[25] Hultcrantz M., Rind D., Akl E.A., Treweek S., Mustafa R.A., Iorio A., Alper B.S., Meerpohl J.J., Murad M.H., Ansari M.T. (2017). The GRADE Working Group clarifies the construct of certainty of evidence. J. Clin. Epidemiol..

[26] Edwards M.A., Roy S. (2017). Academic Research in the 21st Century: Maintaining Scientific Integrity in a Climate of Perverse Incentives and Hypercompetition. Environ. Eng. Sci..

[27] Baethge C., Goldbeck-Wood S., Mertens S. (2019). SANRA—A scale for the quality assessment of narrative review articles. Res. Integr. Peer Rev..

[28] Dickersin K. (1990). The Existence of Publication Bias and Risk Factors for Its Occurrence. JAMA.

[29] Song F., Parekh S., Hooper L., Loke Y.K., Ryder J., Sutton A.J., Hing C., Kwok C.S., Pang C., Harvey I. (2010). Dissemination and publication of research findings: An updated review of related biases. Health Technol. Assess..

[30] Dwan K., Gamble C., Williamson P.R., Kirkham J.J. (2013). The Reporting Bias Group. Systematic Review of the Empirical Evidence of Study Publication Bias and Outcome Reporting Bias—An Updated Review. PLoS ONE.

[31] Turner E.H., Matthews A.M., Linardatos E., Tell R.A., Rosenthal R. (2008). Selective Publication of Antidepressant Trials and Its Influence on Apparent Efficacy. N. Engl. J. Med..

[32] Simmons J.P., Nelson L.D., Simonsohn U. (2011). False-Positive Psychology: Undisclosed Flexibility in Data Collection and Analysis Allows Presenting Anything as Significant. Psychol. Sci..

[33] Gelman A., Loken E. (2014). The Statistical Crisis in Science. Am. Sci..

[34] Ioannidis J.P.A. (2008). Why Most Discovered True Associations Are Inflated. Epidemiology.

[35] Button K.S., Ioannidis J.P.A., Mokrysz C., Nosek B.A., Flint J., Robinson E.S.J., Munafò M.R. (2013). Power failure: Why small sample size undermines the reliability of neuroscience. Nat. Rev. Neurosci..

[36] Riley R.D., Ensor J., Snell K.I.E., Harrell F.E., Martin G.P., Reitsma J.B., Moons K.G.M., Collins G.S., van Smeden M. (2020). Calculating the sample size required for developing a clinical prediction model. BMJ.

[37] Kerr N.L. (1998). HARKing: Hypothesizing After the Results are Known. Pers. Soc. Psychol. Rev..

[38] John L.K., Loewenstein G., Prelec D. (2012). Measuring the Prevalence of Questionable Research Practices With Incentives for Truth Telling. Psychol. Sci..

[39] Banks G.C., Rogelberg S.G., Woznyj H.M., Landis R.S., Rupp D.E. (2016). Editorial: Evidence on Questionable Research Practices: The Good, the Bad, and the Ugly. J. Bus. Psychol..

[40] Greenberg S.A. (2009). How citation distortions create unfounded authority: Analysis of a citation network. BMJ.

[41] Kunda Z. (1990). The case for motivated reasoning. Psychol. Bull..

[42] Nickerson R.S. (1998). Confirmation Bias: A Ubiquitous Phenomenon in Many Guises. Rev. General. Psychol..

[43] Kim S.H., Schramm S., Riedel E.O., Schmitzer L., Rosenkranz E., Kertels O., Bodden J., Paprottka K., Sepp D., Renz M. (2025). Automation bias in AI-assisted detection of cerebral aneurysms on time-of-flight MR angiography. Radiol. Med..

[44] Kocak B., Baessler B., Bakas S., Cuocolo R., Fedorov A., Maier-Hein L., Mercaldo N., Müller H., Orlhac F., Pinto Dos Santos D. (2023). CheckList for EvaluAtion of Radiomics research (CLEAR): A step-by-step reporting guideline for authors and reviewers endorsed by ESR and EuSoMII. Insights Imaging.

[45] Kocak B., Akinci D’Antonoli T., Mercaldo N., Alberich-Bayarri A., Baessler B., Ambrosini I., Andreychenko A.E., Bakas S., Beets-Tan R.G.H., Bressem K. (2024). METhodological RadiomICs Score (METRICS): A quality scoring tool for radiomics research endorsed by EuSoMII. Insights Imaging.

[46] Zwanenburg A., Vallières M., Abdalah M.A., Aerts H.J.W.L., Andrearczyk V., Apte A., Ashrafinia S., Bakas S., Beukinga R.J., Boellaard R. (2020). The Image Biomarker Standardization Initiative: Standardized Quantitative Radiomics for High-Throughput Image-based Phenotyping. Radiology.

[47] Buvat I., Orlhac F. (2019). The Dark Side of Radiomics: On the Paramount Importance of Publishing Negative Results. J. Nucl. Med..

[48] Nardone V., Reginelli A., Rubini D., Gagliardi F., Del Tufo S., Belfiore M.P., Boldrini L., Desideri I., Cappabianca S. (2024). Delta radiomics: An updated systematic review. Radiol. Med..

[49] Traverso A., Wee L., Dekker A., Gillies R. (2018). Repeatability and Reproducibility of Radiomic Features: A Systematic Review. Int. J. Radiat. Oncol. Biol. Phys..

[50] Berenguer R., Pastor-Juan M.D.R., Canales-Vázquez J., Castro-García M., Villas M.V., Mansilla Legorburo F., Sabater S. (2018). Radiomics of CT Features May Be Nonreproducible and Redundant: Influence of CT Acquisition Parameters. Radiology.

[51] Mackin D., Fave X., Zhang L., Fried D., Yang J., Taylor B., Rodriguez-Rivera E., Dodge C., Jones A.K., Court L. (2015). Measuring Computed Tomography Scanner Variability of Radiomics Features. Investig. Radiol..

[52] Larue R.T.H.M., Van Timmeren J.E., De Jong E.E.C., Feliciani G., Leijenaar R.T.H., Schreurs W.M.J., Sosef M.N., Raat F.H.P.J., van der Zande F.H.R., Das M. (2017). Influence of gray level discretization on radiomic feature stability for different CT scanners, tube currents and slice thicknesses: A comprehensive phantom study. Acta Oncol..

[53] Midya A., Chakraborty J., Gönen M., Do R.K.G., Simpson A.L. (2018). Influence of CT acquisition and reconstruction parameters on radiomic feature reproducibility. J. Med. Imaging.

[54] Meyer M., Ronald J., Vernuccio F., Nelson R.C., Ramirez-Giraldo J.C., Solomon J., Patel B.N., Samei E., Marin D. (2019). Reproducibility of CT Radiomic Features within the Same Patient: Influence of Radiation Dose and CT Reconstruction Settings. Radiology.

[55] Zhu L., Dong H., Sun J., Wang L., Xing Y., Hu Y., Lu J., Yang J., Chu J., Yan C. (2024). Robustness of radiomics among photon-counting detector CT and dual-energy CT systems: A texture phantom study. Eur. Radiol..

[56] Zhang H., Lu T., Wang L., Xing Y., Hu Y., Xu Z., Lu J., Yang J., Chu J., Zhang B. (2025). Robustness of radiomics within photon-counting detector CT: Impact of acquisition and reconstruction factors. Eur. Radiol..

[57] Xue C., Yuan J., Lo G.G., Chang A.T.Y., Poon D.M.C., Wong O.L., Zhou Y., Chu W.C.W. (2021). Radiomics feature reliability assessed by intraclass correlation coefficient: A systematic review. Quant. Imaging Med. Surg..

[58] Kendrick J., Francis R.J., Hassan G.M., Ong J.S.L., Jeraj R., Barry N., Ebert M.A. (2025). Deep learning-based PSMA PET segmentation repeatability: A post-hoc analysis of a single-center, prospective, test–retest trial. Radiol. Med..

[59] Whybra P., Zwanenburg A., Andrearczyk V., Schaer R., Apte A.P., Ayotte A., Baheti B., Bakas S., Bettinelli A., Boellaard R. (2024). The Image Biomarker Standardization Initiative: Standardized Convolutional Filters for Reproducible Radiomics and Enhanced Clinical Insights. Radiology.

[60] Orlhac F., Lecler A., Savatovski J., Goya-Outi J., Nioche C., Charbonneau F., Ayache N., Frouin F., Duron L., Buvat I. (2021). How can we combat multicenter variability in MR radiomics? Validation of a correction procedure. Eur. Radiol..

[61] Da-ano R., Masson I., Lucia F., Doré M., Robin P., Alfieri J., Rousseau C., Mervoyer A., Reinhold C., Castelli J. (2020). Performance comparison of modified ComBat for harmonization of radiomic features for multicenter studies. Sci. Rep..

[62] Demircioğlu A. (2025). Reproducibility and interpretability in radiomics: A critical assessment. Diagn. Interv. Radiol..

[63] Parmar C., Rios Velazquez E., Leijenaar R., Jermoumi M., Carvalho S., Mak R.H., Mitra S., Shankar B.U., Kikinis R., Haibe-Kains B. (2014). Robust Radiomics Feature Quantification Using Semiautomatic Volumetric Segmentation. PLoS ONE.

[64] Saha A., Harowicz M.R., Mazurowski M.A. (2018). Breast cancer MRI radiomics: An overview of algorithmic features and impact of inter-reader variability in annotating tumors. Med. Phys..

[65] deSouza N.M., Van Der Lugt A., Deroose C.M., Alberich-Bayarri A., Bidaut L., Fournier L., Costaridou L., Oprea-Lager D.E., Kotter E., Smits M. (2022). Standardised lesion segmentation for imaging biomarker quantitation: A consensus recommendation from ESR and EORTC. Insights Imaging.

[66] Song H., Wang X., Wu R., Liu W. (2024). The influence of manual segmentation strategies and different phases selection on machine learning-based computed tomography in renal tumors: A systematic review and meta-analysis. Radiol. Med..

[67] Poirot M.G., Caan M.W.A., Ruhe H.G., Bjørnerud A., Groote I., Reneman L., Marquering H.A. (2022). Robustness of radiomics to variations in segmentation methods in multimodal brain MRI. Sci. Rep..

[68] Kocak B., Bulut E., Bayrak O.N., Okumus A.A., Altun O., Borekci Arvas Z., Kavukoglu I. (2023). NEgatiVE results in Radiomics research (NEVER): A meta-research study of publication bias in leading radiology journals. Eur. J. Radiol..

[69] Kocak B., Akinci D’Antonoli T., Ates Kus E., Keles A., Kala A., Kose F., Kadioglu M., Solak S., Sunman S., Temiz Z.H. (2024). Self-reported checklists and quality scoring tools in radiomics: A meta-research. Eur. Radiol..

[70] Halligan S., Menu Y., Mallett S. (2021). Why did European Radiology reject my radiomic biomarker paper? How to correctly evaluate imaging biomarkers in a clinical setting. Eur. Radiol..

[71] Bleker J., Yakar D., van Noort B., Rouw D., de Jong I.J., Dierckx R.A.J.O., Kwee T.C., Huisman H. (2021). Single-center versus multi-center biparametric MRI radiomics approach for clinically significant peripheral zone prostate cancer. Insights Imaging.

[72] Doniselli F.M., Pascuzzo R., Mazzi F., Padelli F., Moscatelli M., Akinci D’Antonoli T., Cuocolo R., Aquino D., Cuccarini V., Sconfienza L.M. (2024). Quality assessment of the MRI-radiomics studies for MGMT promoter methylation prediction in glioma: A systematic review and meta-analysis. Eur. Radiol..

[73] Malcolm J.A., Tacey M., Gibbs P., Lee B., Ko H.S. (2023). Current state of radiomic research in pancreatic cancer: Focusing on study design and reproducibility of findings. Eur. Radiol..

[74] Zhong J., Liu X., Lu J., Yang J., Zhang G., Mao S., Chen H., Yin Q., Cen Q., Jiang R. (2025). Overlooked and underpowered: A meta-research addressing sample size in radiomics prediction models for binary outcomes. Eur. Radiol..

[75] Horvat N., Papanikolaou N., Koh D.M. (2024). Radiomics Beyond the Hype: A Critical Evaluation Toward Oncologic Clinical Use. Radiol. Artif. Intell..

[76] Demircioğlu A. (2021). Measuring the bias of incorrect application of feature selection when using cross-validation in radiomics. Insights Imaging.

[77] Kapoor S., Narayanan A. (2023). Leakage and the reproducibility crisis in machine-learning-based science. Patterns.

[78] Demircioğlu A. (2024). Applying oversampling before cross-validation will lead to high bias in radiomics. Sci. Rep..

[79] Marzi C., Giannelli M., Barucci A., Tessa C., Mascalchi M., Diciotti S. (2024). Efficacy of MRI data harmonization in the age of machine learning: A multicenter study across 36 datasets. Sci. Data.

[80] Gidwani M., Chang K., Patel J.B., Hoebel K.V., Ahmed S.R., Singh P., Fuller C.D., Kalpathy-Cramer J. (2023). Inconsistent Partitioning and Unproductive Feature Associations Yield Idealized Radiomic Models. Radiology.

[81] Beddok A., Grogg K., Nioche C., Rozenblum L., Orlhac F., Calugaru V., Crehange G., Shih H.A., Marin T., Buvat I. (2025). Predicting tumor recurrence site after reirradiation in head and neck cancer: A retrospective external validation of a published [18F]-FDG PET radiomic signature. Radiol. Med..

[82] Kocak B., Keles A., Kose F., Sendur A. (2024). Quality of radiomics research: Comprehensive analysis of 1574 unique publications from 89 reviews. Eur. Radiol..

[83] Barry N., Kendrick J., Molin K., Li S., Rowshanfarzad P., Hassan G.M., Dowling J., Parizel P.M., Hofman M.S., Ebert M.A. (2025). Evaluating the impact of the Radiomics Quality Score: A systematic review and meta-analysis. Eur. Radiol..

[84] Spadarella G., Ugga L., Calareso G., Villa R., D’Aniello S., Cuocolo R. (2022). The impact of radiomics for human papillomavirus status prediction in oropharyngeal cancer: Systematic review and radiomics quality score assessment. Neuroradiology.

[85] Van Calster B., McLernon D.J., Van Smeden M., Wynants L., Steyerberg E.W., On behalf of Topic Group ‘Evaluating Diagnostic Tests and Prediction Models’ of the STRATOS Initiative (2019). Calibration: The Achilles heel of predictive analytics. BMC Med..

[86] Vickers A.J., Elkin E.B. (2006). Decision Curve Analysis: A Novel Method for Evaluating Prediction Models. Med. Decis. Mak..

[87] Huang M.L., Ren J., Jin Z.Y., Liu X.Y., Li Y., He Y.L., Xue H.D. (2024). Application of magnetic resonance imaging radiomics in endometrial cancer: A systematic review and meta-analysis. Radiol. Med..

[88] Kocak B., Mese I., Ates Kus E. (2025). Radiomics for differentiating radiation-induced brain injury from recurrence in gliomas: Systematic review, meta-analysis, and methodological quality evaluation using METRICS and RQS. Eur. Radiol..

[89] Cavallo A.U., Ponsiglione A., Pereira B., Di Donna C., Koltsakis E., Vernuccio F., Laudazi M., Cannella R., Fanni S.C., Akinci D’Antonoli T. (2025). CT and MRI radiomics in cardiovascular risk prediction: A systematic review and meta-analysis by the EuSoMII Radiomics Auditing Group. Eur. Radiol..

[90] Yu A.C., Mohajer B., Eng J. (2022). External Validation of Deep Learning Algorithms for Radiologic Diagnosis: A Systematic Review. Radiol. Artif. Intell..

[91] Kocak B., Ponsiglione A., Stanzione A., Ugga L., Klontzas M.E., Cannella R., Cuocolo R. (2024). CLEAR guideline for radiomics: Early insights into current reporting practices endorsed by EuSoMII. Eur. J. Radiol..

[92] Akinci D’Antonoli T., Cuocolo R., Baessler B., Pinto Dos Santos D. (2023). Towards reproducible radiomics research: Introduction of a database for radiomics studies. Eur. Radiol..

[93] Wright B.D., Vo N., Nolan J., Johnson A.L., Braaten T., Tritz D., Vassar M. (2020). An analysis of key indicators of reproducibility in radiology. Insights Imaging.

[94] Ahmadzadeh A.M., Ashoobi M.A., Broomand Lomer N., Elyassirad D., Gheiji B., Vatanparast M., Bathla G., Tu L. (2025). Application of Deep Learning for Predicting Hematoma Expansion in Intracerebral Hemorrhage Using Computed Tomography Scans: A Systematic Review and Meta-Analysis of Diagnostic Accuracy. Radiol. Med..

[95] Zhong J., Lu J., Zhang G., Mao S., Chen H., Yin Q., Hu Y., Xing Y., Ding D., Ge X. (2023). An overview of meta-analyses on radiomics: More evidence is needed to support clinical translation. Insights Imaging.

[96] Nosek B.A., Ebersole C.R., DeHaven A.C., Mellor D.T. (2018). The preregistration revolution. Proc. Natl. Acad. Sci. USA.

[97] Chambers C.D., Tzavella L. (2021). The past, present and future of Registered Reports. Nat. Hum. Behav..

[98] Nosek B.A., Alter G., Banks G.C., Borsboom D., Bowman S.D., Breckler S.J., Buck S., Chambers C.D., Chin G., Christensen G. (2015). Promoting an open research culture. Science.

[99] Scheel A.M., Schijen M.R.M.J., Lakens D. (2021). An Excess of Positive Results: Comparing the Standard Psychology Literature With Registered Reports. Adv. Methods Pract. Psychol. Sci..

[100] Van Griethuysen J.J.M., Fedorov A., Parmar C., Hosny A., Aucoin N., Narayan V., Beets-Tan R.G.H., Fillion-Robin J.C., Pieper S., Aerts H.J.W.L. (2017). Computational Radiomics System to Decode the Radiographic Phenotype. Cancer Res..

[101] Foy J.J., Robinson K.R., Li H., Giger M.L., Al-Hallaq H., Armato S.G. (2018). Variation in algorithm implementation across radiomics software. J. Med. Imaging.

[102] Challa A.B., Radike M., Rizvi A., Weber N.M., Wamil M., Poigai Arunachalam S., Sheedy E., Leng S., Williamson E.E. (2025). Interobserver and intraobserver variability among different vendors for mitral valve assessment: Implications for transcatheter mitral valve repair. Radiol. Med..

[103] Welch M.L., McIntosh C., Haibe-Kains B., Milosevic M.F., Wee L., Dekker A., Huang S.H., Purdie T.G., O’Sullivan B., Aerts H.J.W.L. (2019). Vulnerabilities of radiomic signature development: The need for safeguards. Radiother. Oncol..

[104] Varma S., Simon R. (2006). Bias in error estimation when using cross-validation for model selection. BMC Bioinform..

[105] Moons K.G.M., Damen J.A.A., Kaul T., Hooft L., Andaur Navarro C., Dhiman P., Beam A.L., Van Calster B., Celi L.A., Denaxas S. (2025). PROBAST+AI: An updated quality, risk of bias, and applicability assessment tool for prediction models using regression or artificial intelligence methods. BMJ.

[106] Kocak B., Borgheresi A., Ponsiglione A., Andreychenko A.E., Cavallo A.U., Stanzione A., Doniselli F.M., Vernuccio F., Triantafyllou M., Cannella R. (2024). Explanation and Elaboration with Examples for CLEAR (CLEAR-E3): An EuSoMII Radiomics Auditing Group Initiative. Eur. Radiol. Exp..

[107] Tejani A.S., Klontzas M.E., Gatti A.A., Mongan J.T., Moy L., Park S.H., Kahn C.E., CLAIM 2024 Update Panel (2024). Checklist for Artificial Intelligence in Medical Imaging (CLAIM): 2024 Update. Radiol. Artif. Intell..

[108] Wilkinson M.D., Dumontier M., Aalbersberg I.J.J., Appleton G., Axton M., Baak A., Blomberg N., Boiten J.W., Bonino da Silva Santos L., Bourne P.E. (2016). The FAIR Guiding Principles for scientific data management and stewardship. Sci. Data.

[109] Kocak B., Yardimci A.H., Yuzkan S., Keles A., Altun O., Bulut E., Bayrak O.N., Okumus A.A. (2023). Transparency in Artificial Intelligence Research: A Systematic Review of Availability Items Related to Open Science in Radiology and Nuclear Medicine. Acad. Radiol..

[110] Steyerberg E.W. (2019). Clinical Prediction Models: A Practical Approach to Development, Validation, and Updating [Internet].

[111] Steyerberg E.W., Harrell F.E. (2016). Prediction models need appropriate internal, internal–external, and external validation. J. Clin. Epidemiol..

[112] Huang Y., Li W., Macheret F., Gabriel R.A., Ohno-Machado L. (2020). A tutorial on calibration measurements and calibration models for clinical prediction models. J. Am. Med. Inform. Assoc..

[113] Zhong J., Davey A., Frood R., McWilliam A., Shortall J., Reardon M., Reaves K., Swinton M., Hulson O., West C. (2025). Combining MRI radiomics, hypoxia gene signature score and clinical variables for prediction of biochemical recurrence-free survival after radiotherapy in prostate cancer. Radiol. Med..

[114] Cruz Rivera S., Liu X., Chan A.W., Denniston A.K., Calvert M.J., SPIRIT-AI and CONSORT-AI Working Group (2020). Guidelines for clinical trial protocols for interventions involving artificial intelligence: The SPIRIT-AI extension. Nat. Med..

[115] Liu X., Rivera S.C., Moher D., Calvert M.J., Denniston A.K. (2020). Reporting guidelines for clinical trial reports for interventions involving artificial intelligence: The CONSORT-AI Extension. BMJ.

[116] Chen D., He E., Pace K., Chekay M., Raman S. (2025). Concordance with SPIRIT-AI guidelines in reporting of randomized controlled trial protocols investigating artificial intelligence in oncology: A systematic review. Oncologist.

[117] Chen D., Arnold K., Sukhdeo R., Farag Alla J., Raman S. (2025). Concordance with CONSORT-AI guidelines in reporting of randomised controlled trials investigating artificial intelligence in oncology: A systematic review. BMJ Oncol..

[118] Lekadir K., Frangi A.F., Porras A.R., Glocker B., Cintas C., Langlotz C.P., Weicken E., Asselbergs F.W., Prior F., Collins G.S. (2025). FUTURE-AI: International consensus guideline for trustworthy and deployable artificial intelligence in healthcare. BMJ.

[119] International Medical Device Regulators Forum, Artificial Intelligence/Machine Learning-Enabled Medical Devices Working Group (2025). Good Machine Learning Practice for Medical Device Development: Guiding Principles. IMDRF/AIML WG/N88 FINAL:2025. https://www.imdrf.org/sites/default/files/2025-01/IMDRF_AIML%20WG_GMLP_N88%20Final_0.pdf.

[120] (2017). Regulation (EU) 2017/745 of the European Parliament and of the Council of 5 April 2017 on Medical Devices, Amending Directive 2001/83/EC, Regulation (EC) No 178/2002 and Regulation (EC) No 1223/2009 and Repealing Council Directives 90/385/EEC and 93/42/EEC (Text with EEA Relevance.). OJ L [Internet]. http://data.europa.eu/eli/reg/2017/745/oj.

[121] Park J.E., Kim D., Kim H.S., Park S.Y., Kim J.Y., Cho S.J., Shin J.H., Kim J.H. (2020). Quality of science and reporting of radiomics in oncologic studies: Room for improvement according to radiomics quality score and TRIPOD statement. Eur. Radiol..

[122] Spadarella G., Stanzione A., Akinci D’Antonoli T., Andreychenko A., Fanni S.C., Ugga L., Kotter E., Cuocolo R. (2022). Systematic review of the radiomics quality score applications: An EuSoMII Radiomics Auditing Group Initiative. Eur. Radiol..

[123] Kocak B., Ammirabile A., Ambrosini I., Akinci D’Antonoli T., Borgheresi A., Cavallo A.U., Cannella R., D’Anna G., Díaz O., Doniselli F.M. (2025). Explanation and Elaboration with Examples for METRICS (METRICS-E3): An initiative from the EuSoMII Radiomics Auditing Group. Insights Imaging.

[124] Cavallo A.U., Stanzione A., Ponsiglione A., Trotta R., Fanni S.C., Ghezzo S., Vernuccio F., Klontzas M.E., Triantafyllou M., Ugga L. (2024). Prostate cancer MRI methodological radiomics score: A EuSoMII radiomics auditing group initiative. Eur. Radiol..

[125] Chetan M.R., Gleeson F.V. (2021). Radiomics in predicting treatment response in non-small-cell lung cancer: Current status, challenges and future perspectives. Eur. Radiol..

[126] Ter Maat L.S., van Duin I.A.J., Elias S.G., Leiner T., Verhoeff J.J.C., Arntz E.R.A.N., Troenokarso M.F., Blokx W.A.M., Isgum I., de Wit G.A. (2023). CT radiomics compared to a clinical model for predicting checkpoint inhibitor treatment outcomes in patients with advanced melanoma. Eur. J. Cancer.

[127] Peng W., Wan L., Wang S., Zou S., Zhao X., Zhang H. (2023). A multiple-time-scale comparative study for the added value of magnetic resonance imaging-based radiomics in predicting pathological complete response after neoadjuvant chemoradiotherapy in locally advanced rectal cancer. Front. Oncol..

[128] McGale J., Beddok A., Schwartz L.H., Dercle L. (2026). Radiomics Quality Score 2.0: What changed from version 1.0 and why it matters. Nat. Rev. Clin. Oncol..

[129] Demircioğlu A. (2026). Retractions of publications in radiomics: An underestimated problem?. Eur. Radiol..

[130] Aerts H.J.W.L., Velazquez E.R., Leijenaar R.T.H., Parmar C., Grossmann P., Carvalho S., Bussink J., Monshouwer R., Haibe-Kains B., Rietveld D. (2014). Decoding tumour phenotype by noninvasive imaging using a quantitative radiomics approach. Nat. Commun..

[131] van Timmeren J.E., Cester D., Tanadini-Lang S., Alkadhi H., Baessler B. (2020). Radiomics in medical imaging-“how-to” guide and critical reflection. Insights Imaging.

[132] Lee J., Steinmann A., Ding Y., Lee H., Owens C., Wang J., Yang J., Followill D., Ger R., Mackin D. (2021). Radiomics feature robustness as measured using an MRI phantom. Sci. Rep..

[133] Pavic M., Bogowicz M., Würms X., Glatz S., Finazzi T., Riesterer O., Roesch J., Rudofsky L., Friess M., Veit-Haibach P. (2018). Influence of inter-observer delineation variability on radiomics stability in different tumor sites. Acta Oncol..

[134] Granzier R.W.Y., Verbakel N.M.H., Ibrahim A., van Timmeren J.E., van Nijnatten T.J.A., Leijenaar R.T.H., Lobbes M.B.I., Smidt M.L., Woodruff H.C. (2020). MRI-based radiomics in breast cancer: Feature robustness with respect to inter-observer segmentation variability. Sci. Rep..

[135] Park J.E., Kim H.S., Kim D., Park S.Y., Kim J.Y., Cho S.J., Kim J.H. (2020). A systematic review reporting quality of radiomics research in neuro-oncology: Toward clinical utility and quality improvement using high-dimensional imaging features. BMC Cancer.

[136] Jannot A.S., Agoritsas T., Gayet-Ageron A., Perneger T.V. (2013). Citation bias favoring statistically significant studies was present in medical research. J. Clin. Epidemiol..

[137] Duyx B., Urlings M.J.E., Swaen G.M.H., Bouter L.M., Zeegers M.P. (2017). Scientific citations favor positive results: A systematic review and meta-analysis. J. Clin. Epidemiol..

[138] Vickers A.J., Woo S. (2022). Decision curve analysis in the evaluation of radiology research. Eur. Radiol..

[139] Chiu K., Grundy Q., Bero L. (2017). “Spin” in published biomedical literature: A methodological systematic review. PLoS Biol..

[140] McGrath T.A., McInnes M.D.F., van Es N., Leeflang M.M.G., Korevaar D.A., Bossuyt P.M.M. (2017). Overinterpretation of research findings: Evidence of “spin” in systematic reviews of diagnostic accuracy studies. Clin. Chem..

[141] Oh Y.K. (2026). Position: State-of-the-Art Claims Require State-of-the-Art Evidence. arXiv.

[142] Di Cesare E., Esposito A., Lo Casto A., Mazzei M.A., Polonara G., Sverzellati N., Arrigoni F., Gandolfo N., Miele V., Giovagnoni A. (2025). CT acquisition protocols by pathology, SIRM position paper part 1: Head and neck, brain and spine, chest, cardiovascular. Radiol. Med..

[143] Di Cesare E., Ascenti G., Cappabianca S., Granata C., Reginelli A., Trinci M., Bruno F., Gandolfo N., Miele V., Giovagnoni A. (2025). CT acquisition protocols by pathology, SIRM position paper part 2 (Abdominal and Oncologic Imaging, Urology, Paediatric). Radiol. Med..

[144] Shiroishi M.S., Erickson B.J., Hu L.S., Barboriak D.P., Becerra L., Bell L.C., Boss M.A., Boxerman J.L., Cen S., Cimino L. (2024). The QIBA Profile for Dynamic Susceptibility Contrast MRI Quantitative Imaging Biomarkers for Assessing Gliomas. Radiology.

[145] Sullivan D.C., Obuchowski N.A., Kessler L.G., Raunig D.L., Gatsonis C., Huang E.P., Kondratovich M., McShane L.M., Reeves A.P., Barboriak D.P. (2015). Metrology Standards for Quantitative Imaging Biomarkers. Radiology.

[146] Vickers A.J., Van Calster B., Steyerberg E.W. (2016). Net benefit approaches to the evaluation of prediction models, molecular markers, and diagnostic tests. BMJ.

[147] Park S.H., Han K., Lee J.G. (2024). Conceptual review of outcome metrics and measures used in clinical evaluation of artificial intelligence in radiology. Radiol. Med..

[148] Wagenmakers E.J., Wetzels R., Borsboom D., Van Der Maas H.L.J., Kievit R.A. (2012). An Agenda for Purely Confirmatory Research. Perspect. Psychol. Sci..

[149] Steegen S., Tuerlinckx F., Gelman A., Vanpaemel W. (2016). Increasing Transparency Through a Multiverse Analysis. Perspect. Psychol. Sci..

[150] Simonsohn U., Simmons J.P., Nelson L.D. (2020). Specification curve analysis. Nat. Hum. Behav..

[151] Pai S., Bontempi D., Hadzic I., Prudente V., Sokač M., Chaunzwa T.L., Bernatz S., Hosny A., Mak R.H., Birkbak N.J. (2024). Foundation model for cancer imaging biomarkers. Nat. Mach. Intell..

[152] Moor M., Banerjee O., Abad Z.S.H., Krumholz H.M., Leskovec J., Topol E.J., Rajpurkar P. (2023). Foundation models for generalist medical artificial intelligence. Nature.

[153] Koetzier L.R., Wu J., Mastrodicasa D., Lutz A., Chung M., Koszek W.A., Pratap J., Chaudhari A.S., Rajpurkar P., Lungren M.P. (2024). Generating synthetic data for medical imaging. Radiology.

[154] Mali S.A., Mohammadian Rad N., Woodruff H.C., Depeursinge A., Andrearczyk V., Lambin P. (2025). Harmonizing CT scanner acquisition variability in an anthropomorphic phantom: A comparative study of image-level and feature-level harmonization using GAN, ComBat, and their combination. PLoS ONE.

[155] Floca R., Bohn J., Haux C., Wiestler B., Zöllner F.G., Reinke A., Weiß J., Nolden M., Albert S., Persigehl T. (2024). Radiomics workflow definition & challenges—German priority program 2177 consensus statement on clinically applied radiomics. Insights Imaging.

[156] Santinha J., Pinto Dos Santos D., Laqua F., Visser J.J., Groot Lipman K.B.W., Dietzel M., Klontzas M.E., Cuocolo R., Gitto S., Akinci D’Antonoli T. (2024). ESR Essentials: Radiomics—Practice recommendations by the European Society of Medical Imaging Informatics. Eur. Radiol..

[157] Avanzo M., Soda P., Bertolini M., Bettinelli A., Rancati T., Stancanello J., Rampado O., Pirrone G., Drigo A. (2026). Robust radiomics: A review of guidelines for radiomics in medical imaging. Front. Radiol..

[158] Nosek B.A., Spies J.R., Motyl M. (2012). Scientific Utopia: II. Restructuring Incentives and Practices to Promote Truth Over Publishability. Perspect. Psychol. Sci..

[159] Cagan R. (2013). The San Francisco Declaration on Research Assessment. Dis. Model Mech..

[160] Xu H.L., Gong T.T., Song X.J., Chen Q., Bao Q., Yao W., Xie M.M., Li C., Grzegorzek M., Shi Y. (2025). Artificial Intelligence Performance in Image-Based Cancer Identification: Umbrella Review of Systematic Reviews. J. Med. Internet Res..

[161] Linton-Reid K., Chen M., Boubnovski Martell M., Posma J.M., Aboagye E.O. (2026). Radiomics in clinical radiology: Advances, challenges, and future directions. Clin. Radiol..

[162] European Parliament and Council of the European Union Regulation (EU) 2024/1689 Laying down Harmonised Rules on Artificial Intelligence (Artificial Intelligence Act). Official Journal of the European Union. 12 July 2024; OJ L 2024/1689. https://eur-lex.europa.eu/eli/reg/2024/1689/oj.

[163] U.S. Food and Drug Administration (2024). Marketing Submission Recommendations for a Predetermined Change Control Plan for Artificial Intelligence-Enabled Device Software Functions: Guidance for Industry and FDA Staff.

