# Supplementary material for: Why Radiomics Rarely Reaches the Clinic: Reproducibility, Validation, and Evidence Gap—A Critical Narrative Review
Source: Diagnostics (Basel). 2026 Jul 20;16(14):2266. doi: 10.3390/diagnostics16142266 (PMC13407916; doi:10.3390/diagnostics16142266)
Supplement: Supplementary file 1 [file diagnostics-16-02266-s001.zip › diagnostics-4380561-supplementary.pdf]

Supplementary Material S1

Search strategy

Manuscript: *Why Radiomics Rarely Reaches the Clinic: Reproducibility, Validation and the Evidence Gap — A Critical Narrative Review* (diagnostics-4380561, *Diagnostics*, MDPI).

Q1. Radiomics methodology, measurement reproducibility, and meta-research

("radiomic*"[Title/Abstract] OR "texture analysis"[Title/Abstract]) AND (reproducibility OR repeatability OR robustness OR stability OR standardization OR harmonization OR segmentation OR "feature selection" OR leakage OR "sample size" OR "external validation" OR calibration OR "quality score" OR checklist OR reporting OR meta-research OR "publication bias" OR retraction OR transparency OR "open science")[Title/Abstract]

Q2. Clinical prediction models and imaging AI: validation, clinical utility, and reporting standards

("prediction model" OR "clinical prediction" OR "machine learning" OR "deep learning" OR "artificial intelligence")[Title/Abstract] AND ("external validation" OR calibration OR "decision curve" OR "net benefit" OR "clinical utility" OR "sample size" OR "events per variable" OR "data leakage" OR overfitting OR "cross-validation" OR "risk of bias" OR pitfalls OR TRIPOD OR PROBAST OR CLAIM OR "DECIDE-AI" OR "CONSORT-AI" OR "SPIRIT-AI" OR "FUTURE-AI" OR "reporting guideline")[Title/Abstract]

Q3. Clinical translation, imaging biomarkers, and disease-specific evidence syntheses

("radiomic*" OR "imaging biomarker" OR "quantitative imaging")[Title/Abstract] AND ("clinical translation" OR implementation OR qualification OR "context of use" OR QIBA OR metrology OR "technical performance" OR "added value" OR "clinical utility" OR "systematic review" OR meta-analysis OR prospective)[Title/Abstract]

Q4. Metascience: bias, analytical flexibility, incentives, and evidence appraisal

("publication bias" OR "selective publication" OR "questionable research practices" OR HARKing OR "researcher degrees of freedom" OR "garden of forking paths" OR "analytic flexibility" OR "research waste" OR "perverse incentives" OR "citation distortion" OR "winner's curse" OR "reproducibility crisis" OR replicability OR "motivated reasoning" OR "confirmation bias" OR preregistration OR "registered report" OR "open research" OR "certainty of evidence" OR GRADE OR SANRA)[Title/Abstract]

Q5. Foundation models and generative AI in medical imaging

("foundation model" OR "self-supervised" OR generative OR "synthetic data" OR "generative adversarial")[Title/Abstract] AND ("medical imaging" OR "radiomic*" OR radiology OR "cancer imaging")[Title/Abstract]
